# Taxonomy and Distribution of *Spiraea hypericifolia* in Italy and Typification of the Name *S. flabellata* (Rosaceae)

**DOI:** 10.3390/plants12030536

**Published:** 2023-01-24

**Authors:** Fabio Conti, Fabrizio Bartolucci

**Affiliations:** Centro Ricerche Floristiche dell’Appennino, Parco Nazionale del Gran Sasso e Monti della Laga— Scuola di Bioscienze e Medicina Veterinaria, Università di Camerino, San Colombo, Barisciano, 67021 L’Aquila, Italy

**Keywords:** Central Apennines, Eurasiatic species, lectotype, nomenclature, steppe plant

## Abstract

*Spiraea hypericifolia* is a Eurasiatic species, distributed from SW Europe to C and SW Asia. In Italy, only the alien *S. hypericifolia* subsp. *obovata* was recorded, as naturalized in the Central Apennines. *Spiraea flabellata* was described from Abruzzo (Central Apennines, Italy) by Gussone in 1826 and is currently regarded as a synonym of *S. hypericifolia* subsp. *obovata*. Based on the study of living plants from *locus classicus* and the analysis of the original material traced in BOLO and NAP, *S. flabellata* should be referred instead to *S. hypericifolia* subsp. *hypericifolia*, a taxon reported here for the first time in Italy. The name *S. flabellata* is lectotypified with a specimen kept in NAP. Based on our study, *S. hypericifolia* subsp. *obovata* should be excluded from Italian flora. *Spiraea hypericifolia* subsp. *hypericifolia* should be considered native to Italy and added to the contingent of steppe plants of phytogeographic and conservation interest recorded in the Central Apennines. Finally, the conservation status assessment of *S. hypericifolia* subsp. *hypericifolia* according to IUCN categories and criteria, is proposed and discussed.

## 1. Introduction

As part of the research on the flora of the Abruzzo, Lazio, and Molise National Park [1,2,3], we have studied some critical species groups from a systematic and taxonomic point of view [4,5,6,7]. One of these is *Spiraea hypericifolia* L., a Eurasiatic species with a native range from SW Europe to C and SW Asia. Within *S. hypericifolia* two subspecies are currently recognized in Europe [8]: subsp. *hypericifolia* occurring from SE Europe, Turkey to Siberia, West and Central Himalaya, introduced to Belarus, and subsp. *obovata* (Waldst. & Kit. ex Willd.) H.Huber native in France and Spain, introduced to Belgium and doubtful in Italy and Morocco [9].

*Spiraea flabellata* was described by Gussone [10] from Abruzzo (Central Apennines, Italy) and is currently regarded as a synonym of *S. hypericifolia* subsp. *obovata* [9,11,12,13,14]. According to the Italian checklist [14], *S. hypericifolia* subsp. *obovata* was recorded as a naturalized alien in Abruzzo and a casual alien in Umbria. Other authors report this taxon as doubtfully native to Italy [12,13]. In Abruzzo, it is known for S. Demetrio near L’Aquila, for the surroundings of Trasacco and Fucino Lake ([10,15] as *S. flabellata*), L’Aquila and Teramo ([16] as *S. flabellata*), and recently recorded near Ortucchio (Fucino Plain) ([1] as *S. hypericifolia* subsp. *obovata*). It was also recorded in Umbria near Spoleto and not recently confirmed [15,17,18,19].

The aim of the present work is to clarify the taxonomic identity of *S. flabellata* and to review the taxonomy and distribution of *S. hypericifolia* in Italy.

## 2. Materials and Methods

This study is based mainly on field surveys, on an extensive analysis of the relevant literature, and on careful examination of herbarium specimens (including the original material), kept in APP, AUR, BM, BOLO, H, LINN, LY, NAP, NIM, and TALL (codes follow Thiers [20]). 

In order to investigate the morphological variability of *Spiraea hypericifolia*, 87 dried individuals belonging to *S. hypericifolia* subsp. *hypericifolia* (59) and subsp. *obovata* (24) were studied. According to Dostál [8], the diagnostic characters to identify the two taxa are the shape of leaves, the shape and length of petals in relation to the stamens, and the ratio between the length of sepals and the hypanthium. We measured the characters proposed by Dostál (Table 1) except for the shape and length of petals in relation to stamens. Based on our preliminary observations, these characters are difficult to observe on the herbarium specimens and do not seem to discriminate the infraspecific taxa. The variability of the analyzed morphological characters was described by standard statistical parameters (minimum, maximum, 10^th,^ and 90^th^ percentiles). All morphological characters of herbarium specimens were observed and measured under a Leica MZ16 stereoscopic microscope, using a digital caliper with 0.1 mm precision. The digital images of herbarium specimens were measured with IC Measure version 2.0.0.245. 

The lectotype designation herein follows the Shenzhen Code ([21], hereafter ICN).

## 3. Results

### 3.1. Typification of the Name S. flabellata

***Spiraea flabellata*** Bertol. ex Guss., Pl. Rar. (Gussone): 205 (1826).

Protologue citation: [Italy, Abruzzo] “*In collibus glareosis calcareis Aprutii*; S. Demetrio presso Aquila, e presso Trasacco sul Lago Fucino”.

**Figure 1:**
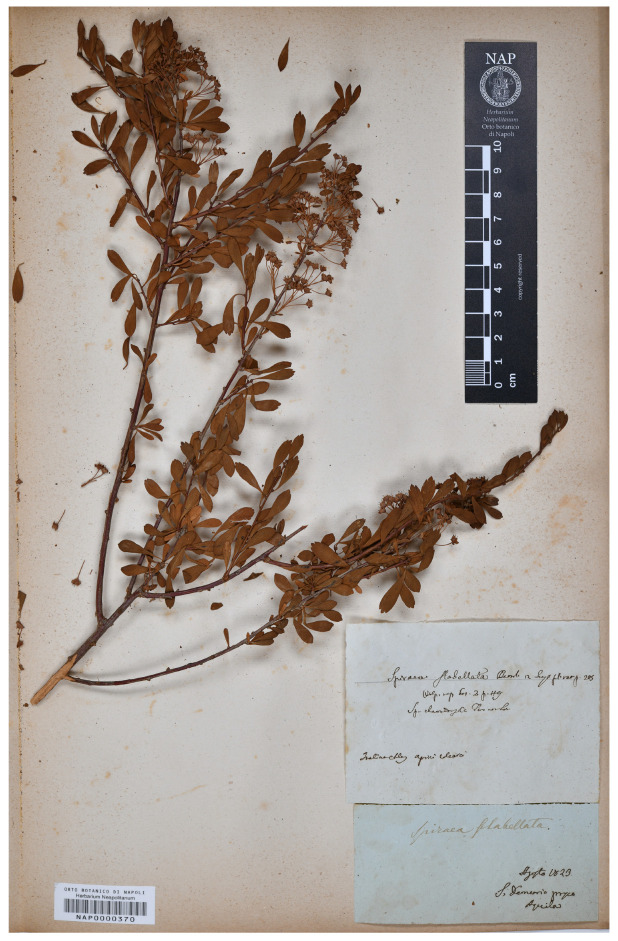
Lectotype of the name *S. flabellata* kept in NAP, barcode NAP0000370 (reproduced with permission of the *Herbarium Neapolitanum*, University of Naples Federico II, Italy).

Lectotype (designated here): [ITALY] S. Demetrio presso Aquila, Agosto 1823, *Gussone s.n.* (NAP barcode NAP0000370 [digital photo!], Figure 1).

**Figure 2:**
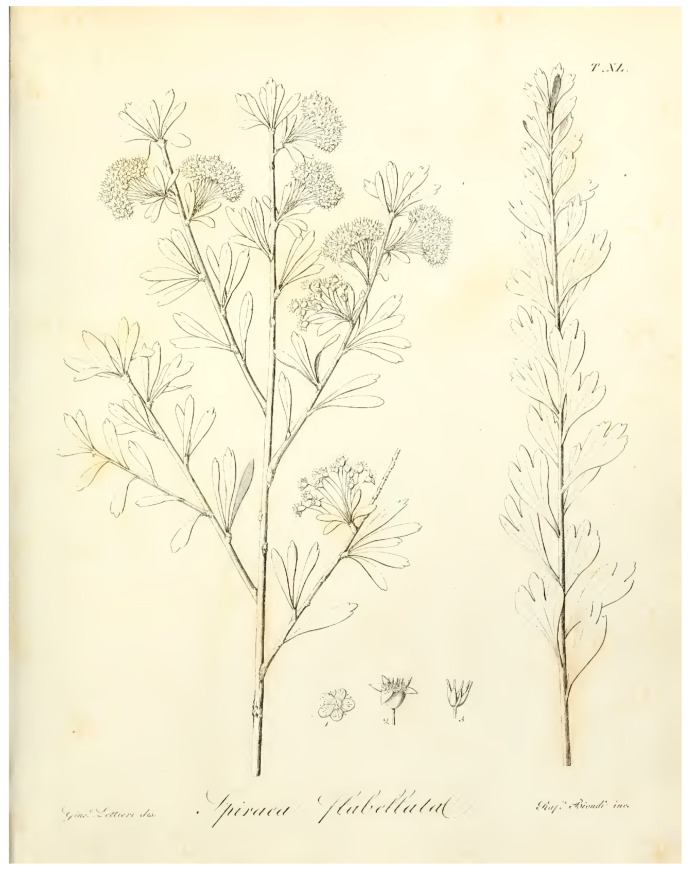
Illustration of *S. flabellata* cited in the protologue by Gussone as “t. 40”.

*Spiraea flabellata* was first described by Gussone [10] from Abruzzo (Central Italy), who provided a detailed description, quoting a precise collection locality and citing an illustration “Ic. nostra t. 40”. We were able to trace some herbarium specimens housed in BOLO and NAP, which can be considered original material (Art. 9.4 of the ICN), as well as the illustration “t. 40” cited in the protologue (Figure 2). In BOLO, we traced one specimen collected by Gussone “Colline presso Aquila a S. Demetrio, misit Gussone 1826”. In the Gussone Herbarium in NAP, we traced four specimens, collected by Gussone: “S. Demetrio presso Aquila, Agosto 1823” (barcode NAP0000370), “S. Demetrio pr. Aquila (barcodes NAP0000371, NAP0000372), “nella Marsica presso il Lago di Fucino a Trasacco, Agosto 1823” (barcode NAP0000373). The herbarium specimen barcoded NAP0000370 is complete, well conserved, agrees with the protologue, and is selected here as lectotype for the name *S. flabellata*.

### 3.2. Taxonomic Treatment

***Spiraea hypericifolia*** L., Sp. Pl. 1: 489 (1753) subsp. ***hypericifolia***

Lectotype (designated by Purohit & Panigrahi 1991: 110). Herb. Linn. No. 651.5 (LINN [digital photo!]), the image of the lectotype is available at https://linnean-online.org/4769/#?s=0&cv=0 (accessed on 10 November 2022). 

= *Spiraea flabellata* Bertol. ex Guss., Pl. Rar. (Gussone): 205 (1826).

Lectotype (see previous pages): [ITALY] S. Demetrio presso Aquila, Agosto 1823, *Gussone* s.n. (NAP barcode NAP0000370 [digital photo!], Figure 1). 

= *Spiraea italica* Raf., New Fl. [Rafinesque] iii. 72 (1838), nom. alt. (Art. 36.3 of the ICN) ≡ *Spiraea reticulata* Raf., New Fl. [Rafinesque] iii. 72 (1838), nom. alt. (Art. 36.3 of the ICN).

Holotype: [illustration] “*Hyperici Spiraea folio*” in Boccone, Museo di Piante Rare della Sicilia, Malta, Corsica, Italia, Piemonte e Germania: Figure in the upper-left corner of pl. 96. 1697.

– *Spiraea chamaedryfolia sensu* Tenore, non L., Fl. Neapol. Prodr. App. IV: 24 (1823), Fl. Napol. 3: IX (1824-1829).

**Figure 3:**
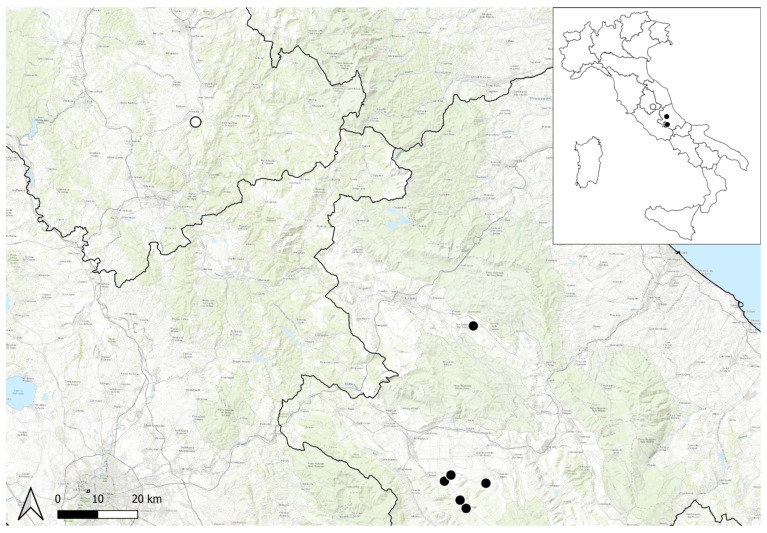
Distribution map of *Spiraea hypericifolia* subsp. *hypericifolia* in Italy, according to the herbarium material studied. Black symbols indicate the population currently present based on field investigations, and empty symbols refer to the old herbarium specimens seen.

Distribution: in Italy, it is currently present only in Abruzzo in the Fucino plain and in the L’Aquila basin (Figure 3). Its presence in Umbria was based on old bibliographic references [15,17,18,19] from hills near Spoleto, and not recently confirmed. We were able to trace an old herbarium specimen housed in BOLO collected from Spoleto (see Specimens examined).

Phenology: flowering from late April to May; fruiting in June and July.

**Figure 4:**
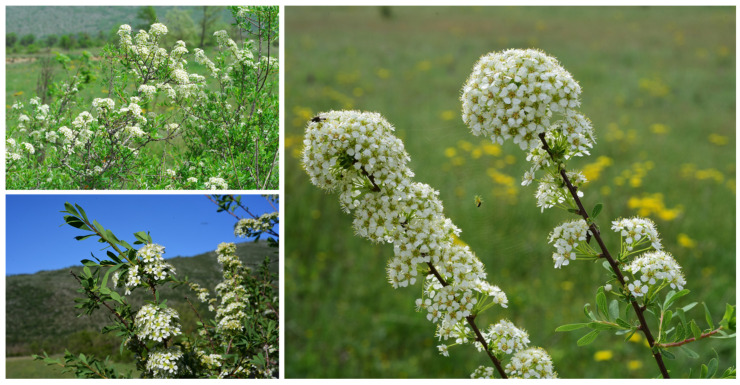
*Spiraea hypericifolia* subsp. *hypericifolia* (Abruzzo, Collelongo, photo by F. Conti).

Habitat and ecology: bushes, hedges in arid hemicryptophytic grasslands at an elevation of 670–900 m a.s.l. We have studied some populations of *S. hypericifolia* in the field of Abruzzo “continental valleys” (including the locus classicus of *S. flabellata*), specifically, in the Fucino plain, near Trasacco and Collelongo, and in the L’Aquila basin between Barisciano and San Demetrio ne’ Vestini (Figure 4). In the latter locality, we were able to confirm the presence of *S. hypericifolia*, where the species was known on the basis of an old report by Gussone [10] and never confirmed again. In the investigated populations, *S. hypericifolia* often forms bushes that are monophytic or accompanied by *Prunus spinosa* L. subsp. *spinosa*, *Crataegus monogyna* Jacq., *Rosa subcollina* (Christ) Vuk., and, more rarely, by *Prunus dulcis* (Mill.) D.A.Webb, surrounded by steppe grasslands. In the Fucino plain, *S. hypericifolia* grows in the fluvio-glacial valley of the Rosa torrent between Trasacco and Villavallelonga, on the edge of arid hemicryptophytic grasslands, rich in steppe relict elements, locally dominated by *Stipa capillata* L. and *Festuca valesiaca* Schleich. ex Gaudin subsp. *valesiaca*, and near Trasacco and Ortucchio. These steppe grasslands have recently been attributed to the priority habitat of EU Habitats Directive “6240 Sub-pannonic steppic grasslands” [22]. The population between Barisciano and S. Demetrio ne’ Vestini, grows in the L’Aquila basin with a marked continental character similar to the Fucino basin, where dominate dry grasslands with *Stipa capillata* and *S. dasyvaginata* Martinovský subsp. *apenninicola* Martinovský & Moraldo.

Conservation status: *Spiraea hypericifolia* currently occurs in Central Apennines in Abruzzo, in the L’Aquila basin, and in the Fucino plain. In the latter locality, the population is partly inside the NATURA 2000 network within the SAC IT7110205 “Parco Nazionale d’Abruzzo”. The population in Umbria (Central Italy), confirmed by one old herbarium specimen, has not been observed for over 180 years. The Extent of Occurrence (EOO) is 231.104 km^2^, and the area of occupancy (AOO) is 24 km^2^ (cell 2 × 2 km) calculated with GeoCAT (Geospatial Conservation Assessment Tool) software [23]. The species is present in two locations (six subpopulations), and a decline in the EOO and AOO was inferred, considering the possible extinction of the population in Umbria. A decline of the quality of habitat, due to the presence of the invasive alien *Ailanthus altissima* (Mill.) Swingle, was observed. According to IUCN [24] criterion B2ab(i,ii,iii,iv), the species is assessed as Endangered (EN) at the regional level (Italy).

Taxonomic notes: based on studied and measured herbarium material, *S. hypericifolia* subsp. *hypericifolia* (including *S. flabellata*) showed narrower leaves than in subsp. *obovata* and hypanthium clearly longer than sepals (see Table 1).

Specimens examined: ***Spiraea hypericifolia*** subsp. ***hypericifolia*.** Europe, Asie et Amerique, 26 December 1900, herb. *R. Bonaparte s.n.* (LY barcode LY0211680, under the name of *S. hypericifolia* var. *acutifolia*); **China**. Westwen Hupe, expedition to China 1907-09, March 1908, *E.H. Wilson 2754* (BM barcode BM001124584, under the name of *S. hypericifolia* var. *hupehensis* ); **Uzbekistan**. Usbeki NSV, Taškendi obl., Bostandõki raj., Bilder-Sai, 2500 m, 25 August 1981, *J. Elliku s.n.* (TALL barcode TALLA007377 ); Taškendi oblast, Bostandõki rajoon, Hudoidot-Sai jõe ääres, 2 September 1981, *J. Elliku s.n.* (TALL barcode TALLA007376); Taškendi oblast, Bostandõki rajoon, Bilder-Sai, ~ 1600 m., 27 August 1981, *J. Elliku s.n.* (TALL barcode TALLA007375); the village of Aktash, 70–80 km NE of Tashkent, above pioneer camp in Karzhantau Ridge of Tien-Shan Mountains, along a stream bank in a rocky canyon, 1700–2000 m, small shrub 1 m. high growing on rocks, 22 July 1986, *T.S. Elias, D. Murray et L. Newcombe 9786* (H barcode H1237319); Taškendi oblast, Abdukarim-Sai, Akbulaki jõe ääres, 2 May 1982, *A. Paivel s.n.* (TALL barcode TALLA007378); **Kyrghyzstan**. Talas region, Talas district, S macroslope of Talas Ala-Too, It-Agar river (left tributary of Chychkan river), gravelly river side deciduous forest, 42.15°N 72.83°E, alt. 1800 m a.s.l., 26 July 2009, *H. Väre 18682* (H QR code C543595); **Russia**. Prov. Samara, distr. Nowo-Usen., in steppes, locis demissioribus, circa p. Walujka, 14-18 May (fl.), 3 June (fr.) 1900, *W. Bogdan s.n.* (H QR code C120069); ibidem, May-June 1900, *W. Bogdan s.n.* (H QR code C120071); Mägi-Altai AO, Ulagani rajoon, Aktaši ümbrus, 6 September 1986, *J. Elliku s.n.* (TALL barcode A007379); regio Volgae inferioris, ripa dextra fluvii Don prope oppidum Surovikino (Russia, pars Europaea, prov. Volgograd), fruticetum stepposum, 6 May 1991, *V. Sagaläev s.n.* (H QR code C120078); Estonia. Viljandi maakond, Õisu, 8 May 1990, *J. Elliku s.n.* (TALL barcode A007384); **Italy**. Spoleto in Umbria, 1834, *Roma Manni s.n.* (BOLO); Ortucchio (L’Aquila), rupi e prati aridi, 680 m, 25 April 1992, *F. Conti s.n.* (APP No. 25542); tra Collelongo e Trasacco (Vallelonga) (Lecce nei Marsi, L’Aquila), mandorleto, prati aridi, 765 m, 19 April 2001, *F. Conti s.n.* (APP Nos. 32699, 32700); Trasacco (L’Aquila), 8 October 2000, *F. Conti s.n.* (APP No. 35199); presso il cimitero di Trasacco (Lecce nei Marsi, L’Aquila), cespuglieti, 700 m, 9 May 2022, *F. Conti, F. Bartolucci s.n.* (APP Nos. 66190–66198, MSMN); *ibidem*, 28 June 2022 *F. Conti, F. Bartolucci s.n.* (APP Nos. 66199, 66200); presso Collelongo (L’Aquila), cespuglieti, 888 m, 9 May 2022, *F. Bartolucci, F. Conti s.n.* (APP Nos. 66201, 66202); Colle Cicogna (Barisciano, L’Aquila), cespuglieti, 845 m, 21 May 2022, *F. Conti s.n.* (APP Nos. 66203–66207). ***Spiraea hypericifolia*** subsp. ***obovata.* France**. Bois de Sauzay à Saint-Loup (Cher), 14 May 1894, *A. Martin s.n.* (LY barcode LY0211200); Rhȏne: Saint Symphorien-sur-Coise, haies, 15 May 1884, *Anthelme s.n.* (LY barcodes LY0211186, LY0211193; AUR barcode AUR03686); Lagrange (Cher), 18 June 1857, … [?] *s.n.* (LY barcode LY0211211); hab. in sylvis petrosis (Berri, Cevennes, Parisiensis), Horti Rhedonensis, *s.c., s.n.* (LY barcode LYJB057021); coteaux calcaires des environs d’Angoulême, 24 April–10 June 1902, *A. Guillon s.n.* (NIM barcode NIME0006457); près Angoulême (Charente), May 1853, *A. Guillon s.n.* (LY barcode LY0211681); chaumes de la Tourette près Angoulême, Charente, 19 April 1859, *A. de Rochebrune s.n.* (LY barcode LY0211677); Le Larzac, à l’Hospitalet (Aveyron), 10 July 1888, *H. Coste s.n.* (AUR barcode AUR03687). **Spain**. Zigoitia, Ondategi, cerro de las Larras (España, prov. Álava), UTM 30 TWN 2154, alt. 580–600 m, orla y claros de quejigal (Quercus faginea) terreno margoso, 25 April 1997, *P.M. Uribe-Echebarría VTT 51413* (H QR code C120115); Berberana (Espagne, prov. Burgos), per après le Puerto de Orduna, au début de la descente vers Berberana, alt. 800 m, platière arbustive, 25 May 1982, *B. De Retz 82984* (H QR code C120116); Barrundia, Urizar. Cerro Arbulo (España, prov. Álava), UTM 30 TWN 3649, alt. 590 m, cerro margoso en zona de quejigal, 9 May 1993, *A.* and *P.M. Uribe-Echebarría VIT 14989* (H QR code C120118); Lumbier (Espagne, Navarra), Foz de Arbayún, bord supérieur de la falaise, coord. 30T XN 5030, alt. 690 m, sur calcaire sec, communautés entre les Ononidetalia et les Rosmarinetalia, endroit fortement venteux, 3 June 1970, *P. Montserrat 1808/70* (H QR code C120117).

## 4. Discussion and Conclusions

On the basis of our study, *Spiraea flabellata* should be considered as a synonym of *S. hypericifolia* subsp. *hypericifolia*, and not a synonym of *S. hypericifolia* subsp. *obovata*, as previously reported [9,11,14]. Accordingly, *Spiraea hypericifolia* subsp. *obovata* should be excluded from Italian flora, while subsp. *hypericifolia* has been reported for the first time in Italy. *Spiraea hypericifolia* subsp. *hypericifolia* is a plant growing in the steppe zone of Europe and Asia. In Italy, it grows in the Central Apennines (Abruzzo, although not confirmed in Umbria) in the internal continental valleys of the Fucino and L’Aquila basins, where the priority habitat of “6240 Sub-pannonic steppic grasslands” has recently been found and a rich contingent of steppic plants of considerable scientific and phytogeographic interest, such as the disjunction with E Europe, was recorded, e.g., *Adonis vernalis* L., *Alyssum desertorum* Stapf., *Androsace maxima* L., *Astragalus exscapus* L. subsp. *exscapus*, *Festuca valesiaca* Schleich. ex Gaudin subsp. *valesiaca*, *Salvia aethiopis* L., and *Stipa capillata* L. [25,26,27,28]. Moreover, some Italian endemics, living in the same areas, must be considered of steppic origin, such as *Goniolimon tataricum* (L.) Boiss. subsp. *italicum* (Tammaro, Pignatti & Frizzi) Buzurović, *Astragalus aquilanus* Anzal. [29,30] and *Adonis fucensis* F.Conti & Bartolucci [31]. The populations of *S. hypericifolia* studied on the field in Abruzzo, characterize communities of shrubs on the edge of fields, pastures, and arid hemicryptophytic grasslands, rich in steppic relict elements, locally dominated by *Stipa capillata*, *S. dasyvaginata* subsp. *apenninicola* and *Festuca valesiaca* subsp. *valesiaca*. Our observations and ecological considerations suggest that *S. hypericifolia*, a typical steppic plant, can be considered native to the Central Apennines. The occurrence of many species of steppe origin and the recently discovered sub-pannonic steppic grasslands confirm the hypothesis that the inner continental valleys of the Central Apennines performed as refugia throughout the Holocene for the late-Pleistocene steppic flora [27].

## Figures and Tables

**Table 1:** Comparison of diagnostic morphological characters based on studied herbarium material between *S. hypericifolia* subsp. *hypericifolia* and *S. hypericifolia* subsp. *obovata*. Characters are reported as 10–90 percentiles (extreme values in brackets).

Characters	*S. hypericifolia* subsp. *hypericifolia*	*S. hypericifolia* subsp. *obovata*
Leaf length (mm)	(8.91)12.21–24.12(28.33)	(9.57)12.59–20.96(23.69)
Leaf width (mm)	(2.86)3.21–5.00(6.43)	(3.96)4.98–9.04(9.69)
Leaf length/leaf width (LL/LW)	(2.86)3.21–5.00(6.43)	(1.69)2.05–2.74(2.90)
Hypanthium length (mm)	(0.86)0.97–2.00(2.46)	(0.86)1.06–1.60(1.74)
Sepal length (mm)	(0.44)0.54–1.40(2.29)	(1.00)1.11–1.58(1.97)
Hypanthium length/sepal length (HL/SL)	(1.07)1.21–1.98(2.02)	(0.75)0.76–1.15(1.19)

## Data Availability

The data presented in the current study are available within the article.
